# Investigation of the effect of rectangular winglet angles on turbulent flow and heat transfer of water/Cu nanofluid in a three-dimensional channel

**DOI:** 10.1016/j.heliyon.2024.e36482

**Published:** 2024-08-18

**Authors:** Mohammad Reza Tavakoli, Omid Ali Akbari, Anoushiravan Mohammadian, Farzad Pourfattah

**Affiliations:** a Department of Mechanical Engineering, Isfahan University of Technology, Isfahan, 84156-83111, Iran; b Southern University Science and Technology, Mechanics and Aerospace Engineering Department, Shenzhen, Guangdong, China

**Keywords:** Heat transfer, Rectangular winglet, Nusselt number, Rectangular channel, Performance evaluation criterion (PEC), Friction coefficient

## Abstract

This numerical simulation studies a homogenous and single-phase nanofluid's turbulent flow and heat transfer behavior in a three-dimensional rectangular microchannel. This study's main purpose is to investigate the use of rectangular winglet angles on flow path and its effect on turbulent flow regime and heat transfer parameters. In the current study, the Reynolds number, winglet attack angle (θ), and twisted angle range (α) (or Pitch angle) from 3000 to 12000, 30°≤ θ ≤ 60°, and 15°≤α ≤ 45°, respectively. Also, Cu nanoparticles with volume fractions of 0–4% are used in water as the base fluid. Results of this study show that heat transfer and flow physics of cooling fluid are affected by the variations of attack angle and winglet twist, and the creation of secondary flows leads to the mixture and deviation of flow. A decrease in the attack angle of the winglet causes the creation of strong vortexes and an increase in Nusselt number and heat transfer. In all investigated situations, with the angle of attack constant, increasing the twist angle can improve the Nusselt number between 11 and 18 percent. Also, increasing the angle of attack of the winglet from 30 to 60° can reduce the Nusselt number by 4–8 percent. The results indicate that changing the winglet angle increases the friction coefficient, and at higher Reynolds numbers, this parameter decreases. Also, by increasing Reynolds number, the ratio of friction coefficient to Nusselt number reduces, leading to the decrease of performance evaluation criterion (PEC).

## 1 Introduction

Recently, many methods have been presented by researchers and industrialists for removing heat flux from heat generation equipment through changing flow patterns with variations of surface geometry. Using twisted tapes and vortex generators to create secondary flows are some examples of these methods. On the other hand, researchers have approved the increase of heat transfer efficiency by using novel fluids, called nanofluids. Combining heat transfer methods in particular industries with higher thermal removal is an efficient method in heat transfer equipment. Researchers have investigated the use of twisted tapes with different structures and vortex generators with special arrangements on flow paths or changing flow regimes with the aim of heat transfer enhancement. Eiamsa-ard et al. [1] experimentally studied turbulent flow hydrodynamics and heat transfer of a tube with delta-winglet twisted tapes at Reynolds numbers of 3000–27000. He concluded that, using vortex generator in tube can improve heat transfer. Wu and Tao [2] three-dimensionally simulated a fin-and-tube heat exchanger with a longitudinal winglet type vortex generator with two angle of attack of 33° and 54° and their numerical results were presented for laminar flow and heat transfer of fin-and-tube surfaces with vortex generators. Colleoni et al. [3] numerically simulated the turbulent flow behavior in a channel with delta winglet VGS and rib-lets. He studied the height of vortex generator and size of ribs and concluded that, using a large and narrow rib and vortex generator results in the highest thermal efficiency. Kamboj et al. [4] investigated CFD simulation and the efficiency of plane and curved trapezoidal winglet type vortex generators. The results of this study showed that curved trapezoidal winglets, compared to the plane type, have better thermal efficiency in laminar and turbulent flow regime. Zhang and Wang [5] numerically studied the fluid flow in Reynolds numbers of 500–700 in a channel with rectangular winglet vortex generator. In this investigation, the effect of the height and attack angle on heat transfer efficiency was studied. They indicated that heat transfer distribution and Nusselt number are related to the secondary flows in different angle of attack and the maximum value of heat transfer is associated with the attack angle of 29°. Min et al. [6] numerically studied the turbulent flow behavior of a new vortex generators arrangement compared to the rectangular winglet type generators and revealed that introduced novel arrangement results in the increase of Nusselt number from 2.1 to 20.7 % and friction coefficient from 4.7 to 104.1 %. Lu and Zhou [7] numerically studied flow and heat transfer inside a channel with plane and curved winglet type vortex generator with punched holes. They showed that vortex generators with curved winglet have higher thermo-hydrodynamic efficiency than the plane type. Also, the punched holes on winglet surface leads to the enhancement of vortex generator efficiency. Sheikhzadeh et al. [8] numerically investigated laminar flow of hybrid Mgo-lethylen glycol (EG) MCNT nanofluid inside a channel with vortex generators with different kinds of winglets at Reynolds numbers of 200–1600. In their study, flow and heat transfer behavior was simulated with three types of rectangular, triangular and trapezoidal vortex generators. Their results presented that rectangular winglet has the highest heat transfer coefficient and channel with triangular and trapezoidal winglets has the highest performance evaluation criterion (PEC). Caliskan et al. [9] experimentally investigated turbulent flow and heat transfer of a nanofluid in a channel with new winglet type vortex generators including the punched triangular and trapezoidal vortex generators. They also changed the winglet angle from 15° to 75° with the changes in the aspect ratio of the channel. They revealed that the thermal efficiency of the surface with a vortex generator is more than the smooth one, and heat transfer increases from 23 to 55 %. The generator with a rectangular winglet has the highest heat transfer. Lin et al. [10] studied laminar flow and mixed heat transfer in a circular tube affected by constant temperature by using twisted tapes and winglets vortex generators with the parallelogram winglet. They studied the changes of geometrical dimensions of twisted tapes as the axial distance (s = 0.83D, 1.0D, 1.25D and 1.67D) and generators angle of attack (α = 14.67°, 21.44° and 27.64°). His results indicated that using twisted tapes and winglets vortex generators are two main factors creating secondary flows and their combination affects flow and heat transfer domain. Also, compared to the smooth tube, tube with parallelogram winglet generator has higher heat transfer. Zhou and Ye [11] experimentally investigated heat transfer, laminar flow and transitional flow regime inside a channel by using vortex generators with a curved trapezoidal winglet pair and showed that this special pattern of vortex generator has the highest PEC. Lin et al. [12] introduced a new fin pattern with curved delta winglet vortex generator and concluded that this new pattern causes better flow motion and reduces the wake region and leads to heat transfer enhancement because of the creation of secondary flows. Akbari et al. [13] numerically studied laminar and turbulent flow and heat transfer of water/Al_2_O_3_ nanofluid at Reynolds numbers of 500–25000 in a three-dimensional tube with twisted tapes with different aspect ratios and concluded that, heat transfer increases with the enhancement of width of twisted tapes and nanoparticles concentration. The existence of solid nanoparticles in base fluid has a great effect on heat transfer in smaller twist ratios (P/W). Other studies on nanofluids in different geometries have indicated transfer enhancement by using these coolants [[14], [15], [16], [17], [18]]. Applying computer science to solve many phenomena has been an effective way to understand the phenomena [19,20]. Computer simulations are very efficient and useful in heat transfer and many studies have been done in this field. Hosseinnezhad et al. [21] numerically studied turbulent flow of water/Al_2_O_3_ nanofluid in a fin and tube heat exchanger with twin twisted tapes insert in a three-dimensional space using finite volume method and indicated that by reducing twisted ratio, increasing volume fraction of nanoparticles in base fluid and disagreement of twisted tapes, average Nusselt number enhances. In twisted ratios of 2.5 and 3.25, the best performance evaluation criteria were 1.6 and 1.55, respectively. Also, in disagree twisted tapes, PEC improvement was significantly more than parallel twisted tapes.

Flow turbulators have applications such as heat exchangers for power plants, cooling systems for oil, chemical and petrochemical industries, food processing and refrigeration industries, industrial. Although many studies have been conducted on the use of flow turbulators, the results of this research can be distinguished by studying the effect of various parameters such as Reynolds number range, winglet angle of attack and the presence of nanofluid in a specific geometry. In this numerical research, turbulent flow and heat transfer of water/Cu nanofluid is three-dimensionally simulated inside a rectangular channel with vortex generators and rectangular winglets using finite volume method at Reynolds numbers of 3000–12000 and winglet angle of attack and twist angles of 30°≤θ ≤ 60° and 15°≤α ≤ 45°, respectively. Presented quantitative results include Nusselt number, temperature domain, friction coefficient and performance evaluation criterion for analyzing flow physics and streamlines.

## 2 Problem statement

**Fig. 1:**
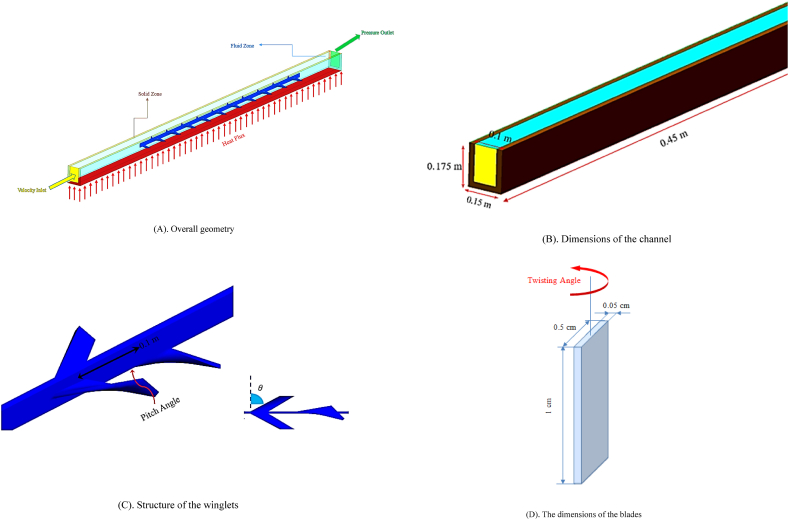
Geometrical schematic and dimensions of studied channel.

This study investigates the passive heat transfer enhancement method by utilizing the blades and complex winglets, which cause circulation and increase turbulent intensity. Water/Cu nanofluid is also selected as a working fluid, and nanoparticle volume fraction ranges between 0 and 4%. In this investigation, 20 aluminum winglets with specific angle of attack and twist angles are located at the center of an aluminum rectangular channel with a thickness of 2.5 cm. The channel wall is under the influence of constant heat flux of q" = 100 kw/m^2^, and the inlet fluid temperature is 300 K. The length of studied channel is 45 cm and the width and height of it are presented in Fig. 1 (A, B, C & D). Figure (1-A) shows the overall geometry, Figure (1-B) shows the dimensions of the channel, Figure (1-C) shows the structure of the winglets, and Figure (1-D) shows the dimensions of the blades.

Studied geometry includes three parts. The first part (length of 10 cm) is the developed flow length that is smooth and has no winglets. Insulated winglets are located in the middle part (length of 20 cm), and the rest part of the channel is smooth with no winglets. The length, pitch, width, and thickness of winglets in the latitude direction are 1 cm, 10 cm, 0.5 cm, and 0.05 cm, respectively. In this study, the angles of twisted winglets include the angle of the winglet perpendicular to the flow direction, called attack angle (θ), and the twisted angle of the head of twisted winglets, called twist angle (α). In the current research, the changes of geometrical parameters such as attack angle and twist angle of winglets are 30°≤ θ ≤ 60° and 15°≤α ≤ 45°, respectively. Turbulent flow is investigated in a three-dimensional space at Reynolds numbers of 3000–12000 and 0–4% Cu nanoparticles volume fractions using finite volume method. In this numerical simulation, nanofluid properties are constant with temperature and no-slip boundary condition is applied to the solid walls. Flow is three-dimensional, steady, incompressible and turbulent. Volume forces and radiational heat transfer are negligible. Also, the cooling nanofluid is a Newtonian and single phase.

## 3 Governing equations

### 3.1 Governing equation on turbulent flow

Governing equations on turbulent flow and heat transfer domain in a rectangular channel are continuity equation, momentum equation and energy consistency equation in three-dimensional Cartesian coordinates presented as follows [22]. Also, k-є sst model is used for simulating turbulent flow and heat transfer as follow:

Equation (1) continuity equation:

(1) $$\frac{\partial }{\partial X_{i}}\left(\rho \;u_{i}\right)=0$$

Equation (2) momentum equation:

(2) $$\frac{\partial }{\partial X_{j}}\left(\rho \;u_{i}\;u_{j}\right)=-\frac{\partial P}{\partial X_{i}}+\frac{\partial }{\partial X_{j}}\left[\mu \left(\frac{\partial u_{i}}{\partial X_{j}}+\frac{\partial u_{j}}{\partial X_{i}}-\frac{2}{3}\delta_{ij}\frac{\partial u_{i}}{\partial X_{j}}\right)\right]+\frac{\partial }{\partial X_{j}}\left(-\rho \;\bar{u_{i}^{/}\;u_{j}^{/}}\right)$$

Equation (3) energy equation:

(3) $$\frac{\partial }{\partial X_{i}}\left(u_{i}\left(E\rho +P\right)\;\right)=\frac{\partial }{\partial X_{j}}\left[\left(\lambda +\frac{Cp\mu_{t}}{{\mathrm{Pr}}_{t}}\right)\frac{\partial T}{\partial X_{j}}+u_{i}{\left({\tau_{i}}_{j}\right)}_{eff}\right]=0$$

where E is total energy and (τ_ij_)_eff_ is the stress tensor deviation defined as equations (4), (5) [23]:

(4) $$E=CpT-\left(P/\rho \right)+\left(u^{2}/2\right)$$

(5) $${\left(\tau_{ij}\right)}_{eff}=\left[\mu_{eff}\left(\frac{\partial u_{j}}{\partial X_{i}}+\frac{\partial u_{i}}{\partial X_{j}}\right)-\frac{2}{3}\mu_{eff}\frac{\partial u_{i}}{\partial X_{j}}\delta_{ij}\right]$$

Transport equation for shear stress transport k-є model is as follow [24]:

(6) $$\frac{\partial }{\partial X_{i}}\left(\rho \;k\;u_{i}\right)=\frac{\partial }{\partial X_{j}}\left(\Gamma_{k}\frac{\partial k}{\partial X_{j}}\right)+G_{k}-Y_{k}+S_{k}$$

(7) $$\frac{\partial }{\partial X_{i}}\left(\rho \omega \;k\;u_{i}\right)=\frac{\partial }{\partial X_{j}}\left(\Gamma_{\omega }\frac{\partial \omega }{\partial X_{j}}\right)+G_{\omega }-Y_{\omega }+D_{\omega }+S_{\omega }$$

where in equation (6), G_k_ is the generated turbulent kinetic energy due to the average velocity gradient and in equation (7) G_w_ indicates the generation of this term from ω.

Also, at the interface of solid-fluid, we have v = 0, w = 0, u = 0, and T_s_ = T_f_ while also (Equation (8)):

(8) $$K_{s}\frac{\partial T_{s}}{\partial n}=K_{t}\frac{\partial T_{s}}{\partial n}$$

and for the bottom wall of the bottom channel ${q_{w}}^{\prime \prime }=-K_{s}\frac{\partial T}{\partial x}=cte$. For the remaining walls, the insulation boundary condition is given by $\frac{\partial T_{s}}{\partial n}=0$.

### 3.2 Equations related to the measured parameters

For determining heat transfer and flow hydrodynamics, following definitions are used:

Friction coefficient is one of the parameters used for studying the hydrodynamic performance of channel which can be calculated according to equation (9) [23]:

(9) $$f=2\Delta P\frac{D_{h}}{L}\frac{1}{\rho {u_{in}}^{2}}$$

In equation (8), D_h_ is the hydraulic diameter of channel which equals with channel height (H) and L, ρ and u_in_ are length, density and inlet velocity of flow, respectively. Average Nusselt number (Equation (10)) is obtained as follow [23]:

(10) $$Nu_{ave}=\frac{q^{\prime \prime }D_{h}}{k_{f}\left(T_{w}-T_{m}\right)}$$

In equation (9), T_w_ and T_m_ are microchannel wall temperature and average bulk temperature, respectively. For general evaluation of performance evaluation criterion of indented microchannel compared to smooth one, PEC parameter is defined as the performance evaluation criterion according to equation (11) [23]:

(11) $$PEC=\frac{\left(\frac{Nu_{ave}}{Nu_{ave,s}}\right)}{{\left(\frac{f}{f_{s}}\right)}^{\left(1/3\right)}}$$

The amount of total entropy, which includes the increase in entropy due to heat transfer and flow friction, is calculated from equation (12).

(12) $$S_{\text{gen}}=\frac{k_{\text{eff}}}{{\bar{T}}^{2}}\left[{\left(\frac{\partial \bar{T}}{\partial x}\right)}^{2}+{\left(\frac{\partial \bar{T}}{\partial y}\right)}^{2}\right]+\frac{\mu_{\text{eff}}}{\bar{T}}\left\{2\left[{\left(\frac{\partial \bar{u}}{\partial x}\right)}^{2}+{\left(\frac{\partial \bar{v}}{\partial y}\right)}^{2}\right]+{\left(\frac{\partial \bar{u}}{\partial x}+\frac{\partial \bar{v}}{\partial y}\right)}^{2}\right\}$$

### 3.3 Introducing nanofluid properties

In this paper, water/Cu nanofluid is proposed as incompressible and single phase with constant thermophysical properties. Physical properties are computed according to the volume fraction of nanoparticles and inlet temperature of fluid. Density, specific thermal capacity of nanofluid, the ratio of nanofluid viscosity to base fluid and the ratio of nanofluid thermal conductivity to base fluid are indicated in Table (1) [25].

**Table 1:** Thermophysical properties of nanofluid in different volume fractions.

Thermo-physical properties	water	Cu	φ = 0.02	φ = 0.04
ρ (kg/m^3^)	997.1	8933	1156	1315
C_p_ (J/kg.K)	4179	385	3593	3148
k (W/m K)	0.613	401	0.65	0.69
μ (N.s/m^2^)	8.91′10^−4^	–	11.987′10^−4^	14.932′10^−4^

## 4 Numerical procedure and assumptions

The current numerical procedure domain is considered in a three-dimensional Cartesian space and turbulent regime. In this simulation, the finite volume method is used. The SIMPLE algorithm is employed for coupling pressure and velocity domain, and for discretizing the equations, the second-order upwind method is utilized. The maximum residual for this simulation is considered 10^−6^. In this research, nanofluid flow is turbulent, single-phase, three-dimensional, steady, and Newtonian, and the radiation effects are negligible. Also, the thermophysical properties of nanofluid are constant with temperature. Finally, small gridding (y_t_ ≤ 1) is used in regions close to the solid walls due to the turbulent flow regime.

### 4.1 Grid convergence Index (GCI)

**Fig. 2:**
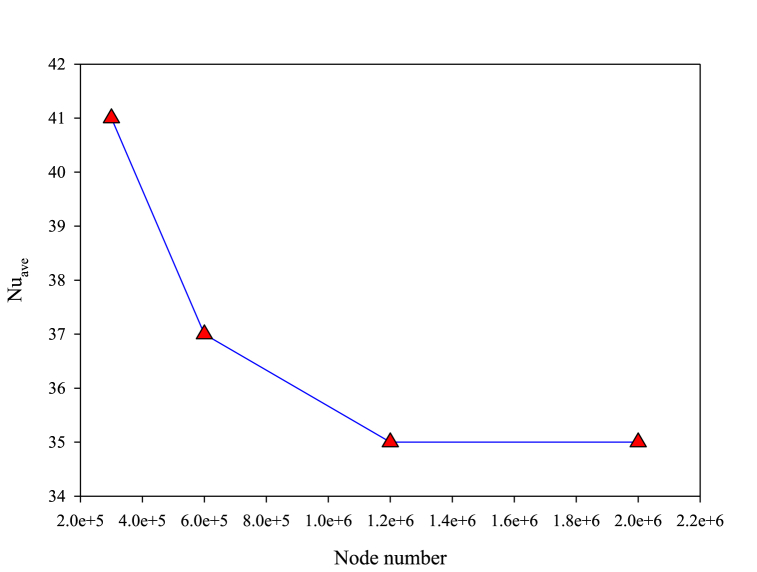
Investigation of **GCI** in present study.

For investigating **GCI**, Nusselt number is studied in various element numbers ranging from 300000 to 2000000 at maximum Reynolds number, pitch and attack angle (Fig. 2). According to Figure (2), in element number of 1200000 obtained results are independent from grid number and in all investigated states, this element number is used.

### 4.2 Validation

**Fig. 3:**
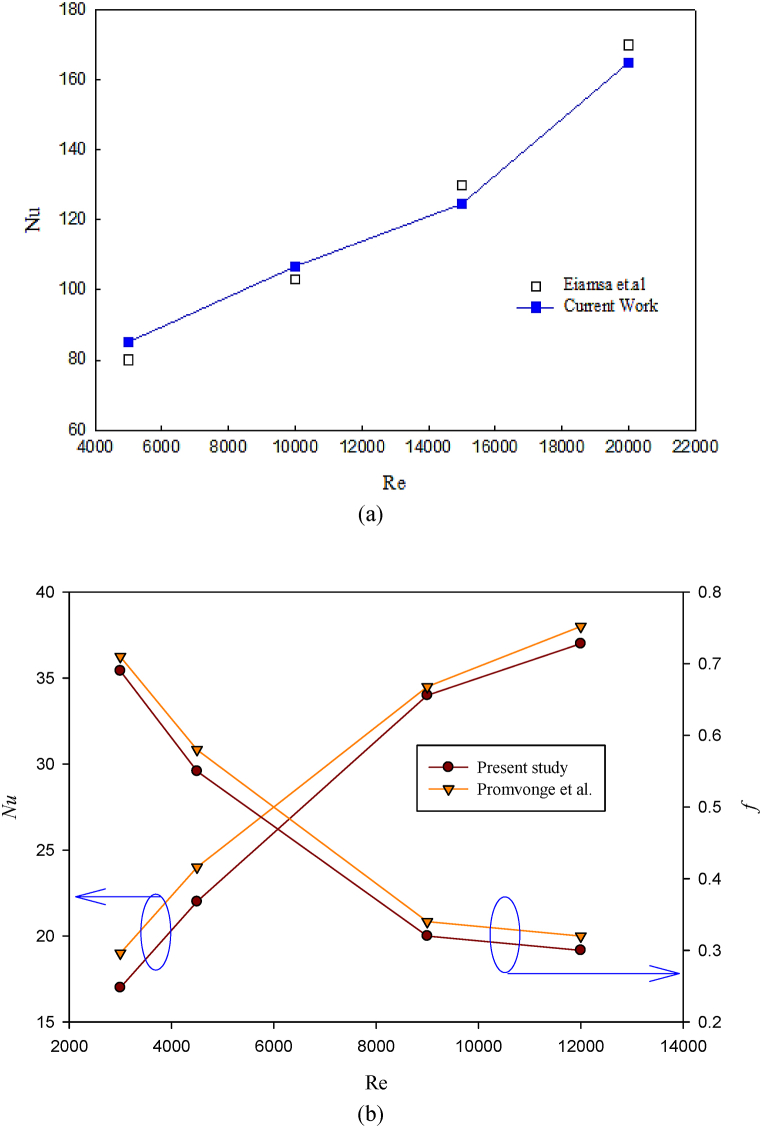
Validation of present numerical results with (a) Eiamsa-Ard et al. [26] and (b) Promvonge et al. [27].

To ensure the accuracy of the numerical procedure, it is necessary to compare the numerical results with other experimental results in a similar field. Figure (3-a) compares the Nusselt number with the reference results Eiamsa-Ard et al. [26]. As can be seen, the obtained numerical results are in agreement with the experimental results of Eiamsa-Ard et al. [26] (maximum error is 5 %), showing that the present numerical procedure is accurate. On the other hand, the validity of the present study has also been examined with the relationships extracted from the experimental results of Promvonge et al. [27]. This research was done to determine the Nusselt number and friction coefficient factors as $Nu=0.108{\text{Re}}^{0.786}\;{\mathrm{Pr}}^{0.4}{R_{B}}^{0.135}{R_{P}}^{-0.097}$ and $f=0.744{\text{Re}}^{-0.001}{R_{B}}^{0.439}{R_{P}}^{-0.271}$, respectively, and for the Reynolds number range of 3000–12000. In the behavior of the figures in Figure (3-b), the validity of the obtained results with the values obtained from the experimental relations shows the accuracy of the results obtained from this simulation.

### 4.3 Boundary conditions

This study applies the finite volume method for numerical simulation in a three-dimensional space. The studied geometry is a rectangular aluminum channel. In all investigated cases, fluid flow is simulated in a turbulent regime. In studied geometry, the inlet velocity boundary condition is applied at the inlet section, and the outlet pressure boundary condition is used at the outlet section of the channel. The studied channel is under the influence of constant heat flux only at the bottom region. Also, other walls of the channel are insulated. At the outlet region, the developed condition is established; therefore, axial velocity and temperature changes are zero.

## 5 Results and discussions

The current study investigates the flow field and heat transfer characteristics of water/Cu nanofluid (volume fractions of 0–6%) in the channel. Simulation of the numerical solving domain includes a rectangular channel with twisted winglets at the center of the channel. The primary purpose of this investigation is to study the effect of twisted winglet type with different angle of attack and twist angles on flow mixture for a limited number of winglets on the latitude direction of flow. Obtained results are plotted as the average temperature domain, Nusselt number, friction coefficient and performance evaluation criterion.

### 5.1 Nusselt number behavior

**Fig. 4:**
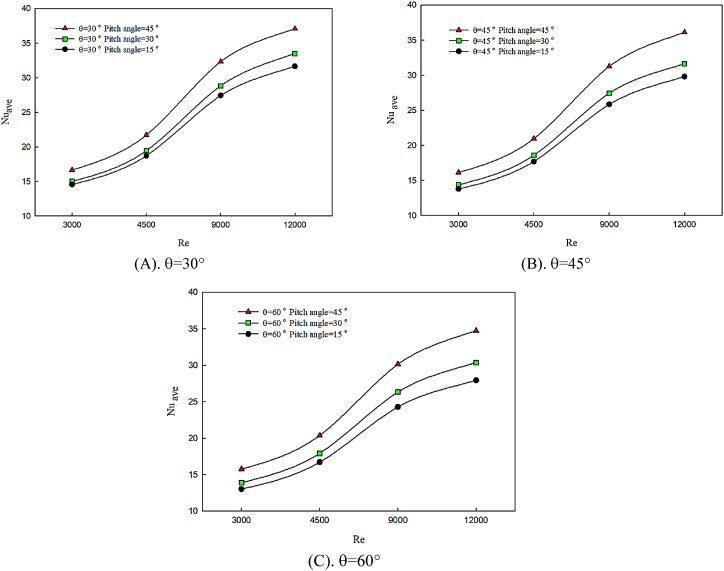
The effect of twist angle of winglet on average Nusselt number in constant attack angle.

In Fig. 4 (A, B & C), the effects of the variations of the twist angle of the winglet on average Nusselt number behavior are presented on the hot wall of the channel in angle of attack of θ = 30° (Figure (4-A)), θ = 45° (Figure (4-B)), and θ = 60° (Figure (4-C)). This study investigates in twist angles of α = 15°, α = 30° and α = 45° for pure water fluid. Any factor disturbing the bulk of fluid and increasing the mixing of the flow leads to the motion of hot fluid in the central region of the channel. Also, the presence of insulated walls results in better temperature distribution among fluid layers. Therefore, heat transfer and temperature exchange between cold fluid and hot surface increase and temperature gradients reduce. According to Figure (4), the changes in winglet attack angle affect the intensity and power of created longitudinal vortexes, and by decreasing the attack angle of the winglet, the created vortexes become stronger. Winglet blocks fluid motion and by colliding with winglet and creating strong vortexes, due to the constriction of section, fluid has higher heat transfer. Hence, the average Nusselt number enhances, and the cooling performance of the hot surface improves. By increasing the Reynolds number, due to the momentum enhancement of fluid, the creation of secondary flows in higher twist angles is considerable on the latitude direction of flow, which leads to Nusselt number enhancement. On the other hand, by decreasing the attack angle, twist angle augmentation has a better effect on average Nusselt number enhancement.

**Fig. 5:**
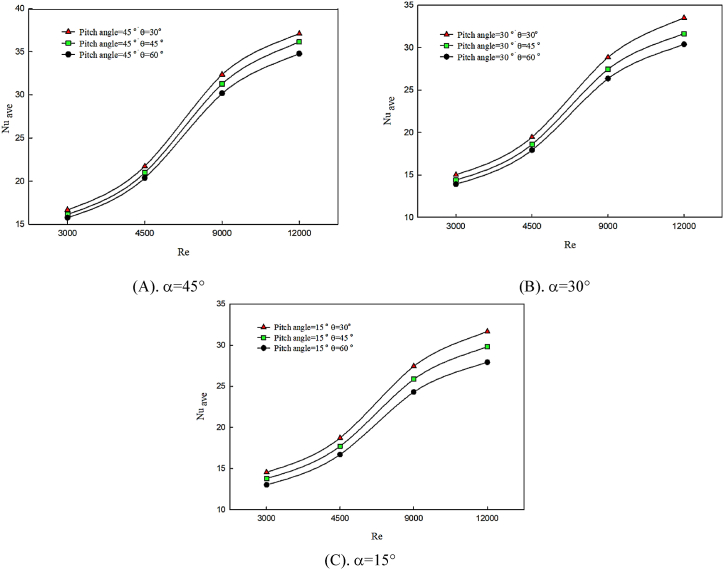
The effect of attack angle on average Nusselt number in constant twist angle.

Fig. 5 (A, B & C) compares the effect of the variations of winglet attack angle on average Nusselt number in the twist angles of α = 45° (Figure (5-A)), α = 30°(Figure (5-B)), and α = 15° (Figure (5-C)), on the hot wall of the channel. Also, the effect of Reynolds number changes on the mentioned factors is explained for each twist angle. The changes in the winglet attack angle affect the intensity and power of created longitudinal vortexes, and by decreasing the attack angle of the winglet, the created vortexes become stronger. Winglet blocks fluid motion and by colliding with winglet and creating strong vortexes, due to the constriction of section, fluid has higher heat transfer. Therefore, in an attack angle of 30°, the average Nusselt number has a significant value compared to other angle of attack. On the other hand, twist angle enhancement caused by attack angle changes has an insignificant effect on Nusselt number. Increasing the twisted angle of the winglet at higher Reynolds numbers and small angle of attack of the winglet has a considerable effect on heat transfer and Nusselt number enhancement.

### 5.2 Average temperature of hot wall

**Fig. 6:**
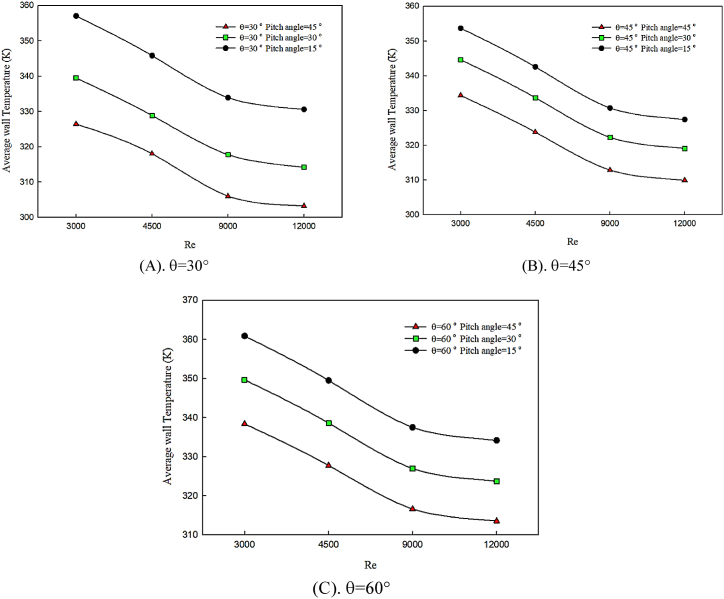
The effect of twist angle on average temperature of wall.

In Fig. 6 (A, B & C), the average temperature changes of the hot wall of the channel with the increase of the twist angle of the winglet are presented for the twist angles of α = 15°, α = 30°, and α = 45°, in θ = 30° (Figure (6-A)), θ = 45° (Figure (6-B)), and θ = 60° (Figure (6-C)). In all studied graphs, the behavior of pure water fluid in constant attack angle is compared for Reynolds numbers of 3000–12000. Since the hot surface is affected by constant heat flux and temperature gradients are always created on flow direction, the created thermal boundary layer significantly grows along the smooth surface of the channel. Flow mixture factors can disturb the thermal boundary layer and decrease temperature gradients. The twist angle of the winglet causes flow deviation and a complete mixture of flow with fluid layers close to the hot surface. Therefore, by increasing the twist angle of the winglet, the flow mixture decreases, which leads to the decreased amplitude of created oscillations on cooling fluid direction on a hot surface. Hence, by augmenting the winglet's twist angle, the hot surface's temperature increases, which has no effect on heat transfer improvement.

On the other hand, by increasing the attack of angle of the winglet, the flow mixture reduces, and the variations of the average temperature of the hot surface are enhanced. Therefore, heat transfer decreases. The considerable point is that, by increasing the angle of attack of the winglet, average temperature graphs have similar quantitative values, indicating that a smaller angle of attack of a twisted winglet has a significant effect on temperature distribution and preservation of temperature gradients. Also, by enhancing the Reynolds number, the hot wall's average temperature significantly decreases, leading to the decreasing trend of temperature graphs with Reynolds number changes.

**Fig. 7:**
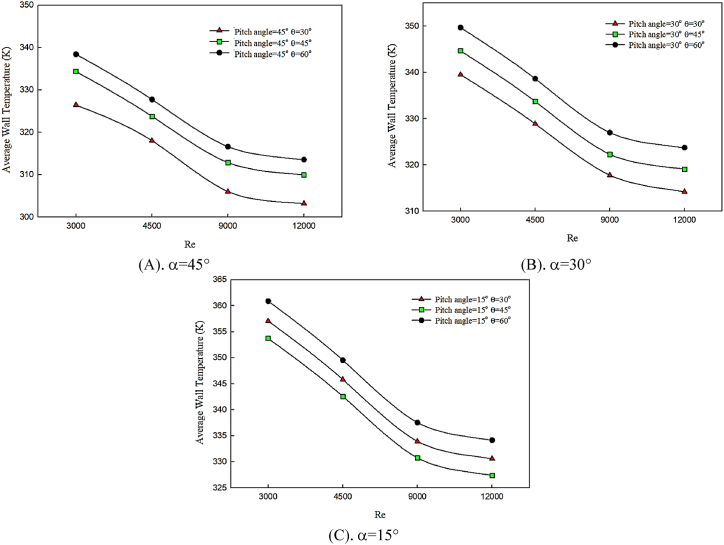
The effect of twist angle on average temperature of wall in constant attack angle.

In Fig. 7 (A, B & C), the behavior of the average temperature of the hot wall with the changes of twist angle is presented for constant angle of attack of θ = 30°, θ = 45°, and θ = 60°, in α = 45° (Figure (7-A)), α = 30° (Figure (7-B)), and α = 15° (Figure (7-C)). By increasing vortex strength on flow direction, the mixture of fluid layers is uniformly distributed among different regions, including hot and insulated boundaries. Also, Reynolds number enhancement improves heat transfer and decreases the temperature domain of fluid and hot surface. By reducing the angle of attack and disturbing the thermal boundary layer, better temperature distribution is obtained along the channel and flow, which is affected by the twist angle of the winglet, which deviates and collides with the hot surface. According to the graphs, the best temperature distribution on a hot surface is related to the maximum Reynolds number. Compared to the other studied cases, the average temperature of the hot surface decreases by decreasing the angle of attack and increasing the twist angle.

### 5.3 Friction coefficient changes

**Fig. 8:**
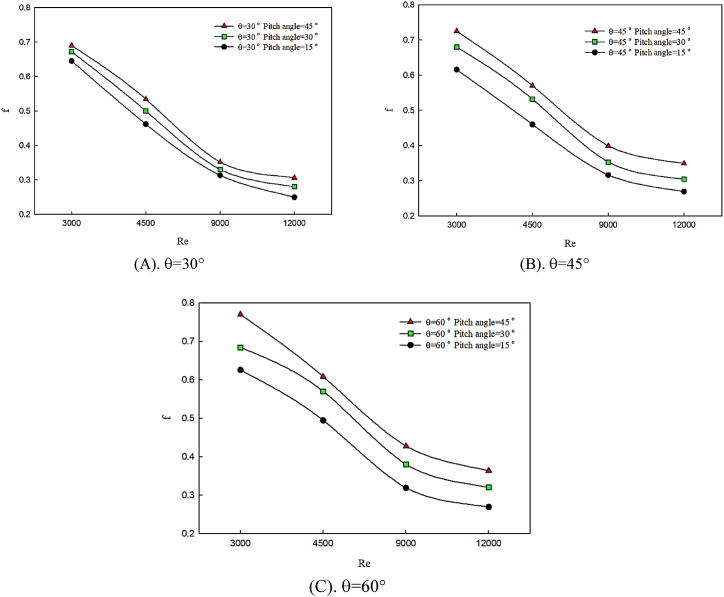
The effects of twist angle changes on Moody friction coefficient.

Fig. 8 (A, B & C) indicates the effect of the changes in the twist angle of the winglet in θ = 30° (Figure (8-A)), θ = 45° (Figure (8-B)), and θ = 60° (Figure (8-C)), on Moody friction coefficient behavior. In this figure, by increasing the attack angle of the winglet, latitudinal changes are created in flow direction, and by changing the twist angle of the winglet, the flow mixture is enhanced. Some parameters such as the changes of attack angle may lead to a temporary blockage on flow direction and pressure drop along channel. The increase of friction coefficient with the enhancement of twist angle is due to the flow deviation caused by the curved flow direction and collusion with the winglets. Hence, created secondary flows are augmented and vortexes are created. Also, big changes in the flow path leading to a decrease in fluid momentum and an increase in pressure drop. By increasing attack angle of winglet from 30° to 45° and twist angle of winglet, friction coefficient enhances. It can be said that, in an angle of attack of 30°, compared to other investigated angle of attack, the changes in friction coefficient are minimal.

On the other hand, Reynolds number enhancement leads to the increase of axial velocity components, which decreases the effect of pressure drop caused by flow motion among winglets. Also, variations of twist angle in the minimum angle of attack have less influence on friction coefficient enhancement at lower Reynolds numbers than other investigated Reynolds numbers and attack angles. Therefore, by increasing the angle of attack, friction coefficient variations are enhanced.

### 5.4 Performance evaluation criterion (PEC)

**Fig. 9:**
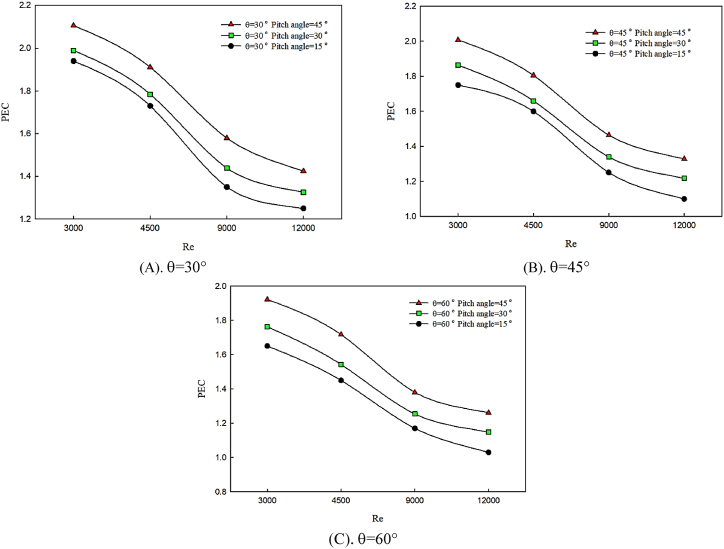
Changes of performance evaluation criterion (PEC) with the changes of twist angle.

Fig. 9 (A, B & C) compares the changes in performance evaluation criterion (PEC) with the variations of the twist angle of the winglet in terms of the Reynolds number for θ = 30° (Figure (9-A)), θ = 45° (Figure (9-B)), and θ = 60° (Figure (9-C)). As it is seen, changes of angle of attack and twist angle of winglet affected heat transfer rate and pressure loss. Based on Fig. 9 (A, B & C), the performance evaluation criterion (PEC) shows a decreasing trend with the increasing the Reynolds number. Also, by increasing twist angle of winglet, heat transfer enhances which results in the increase of fluid momentum depreciation and dominancy of friction coefficient behavior. According to the performance evaluation criterion (PEC), the existence of winglets leads to heat transfer augmentation, however, fluid momentum deprecation enhances, therefore, at high Reynolds numbers using this method for increasing performance evaluation criterion (PEC) is not recommended.

### 5.5 Variations of flow and heat transfer parameters by using nanofluid

**Fig. 10:**
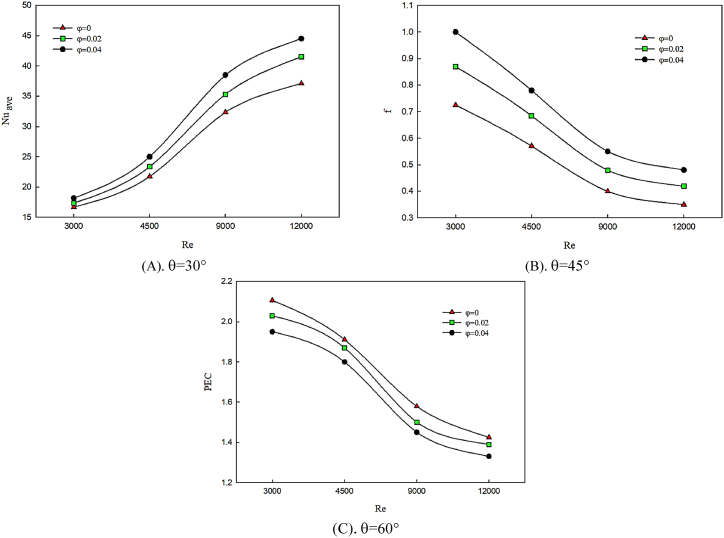
The effect of attack angle changes on performance evaluation criterion in twist angle of 45° and attack angle of 30° for different nanoparticles volume fractions.

Fig. 10 (A, B & C) presents the effect of attack angle changes on Nusselt number, friction coefficient and performance evaluation criterion (PEC) by using nanofluid with different volume fraction of nanoparticles. According to Fig. 10 (A, B & C), twist angle is constant and the value of PEC is compared at Reynolds numbers of 3000–12000 and angle of attack of θ = 30° (Fig. (10-A)), θ = 45° (Fig. (10-B)) and θ = 60° (Fig. (10-C)). The existence of nanoparticles in cooling fluid results in heat transfer enhancement because of the augmentation of thermal conductivity and uniform temperature distribution in studied geometry. On the other hand, solid nanoparticles lead to the increase of density and viscosity of cooling fluid and higher fluid momentum depreciation while moving among the winglets. Twist angle changes are more obvious in the trend of friction coefficient enhancement. According to the effect of the changes of Nusselt number and friction coefficient on performance evaluation criterion, although thermal conductivity augmentation of nanofluid by adding nanoparticles is efficient, the considerable value of friction coefficient leads to the reduction of performance evaluation criterion (PEC) which is obvious at higher Reynolds numbers.

### 5.6 Flow velocity

**Fig. 11:**
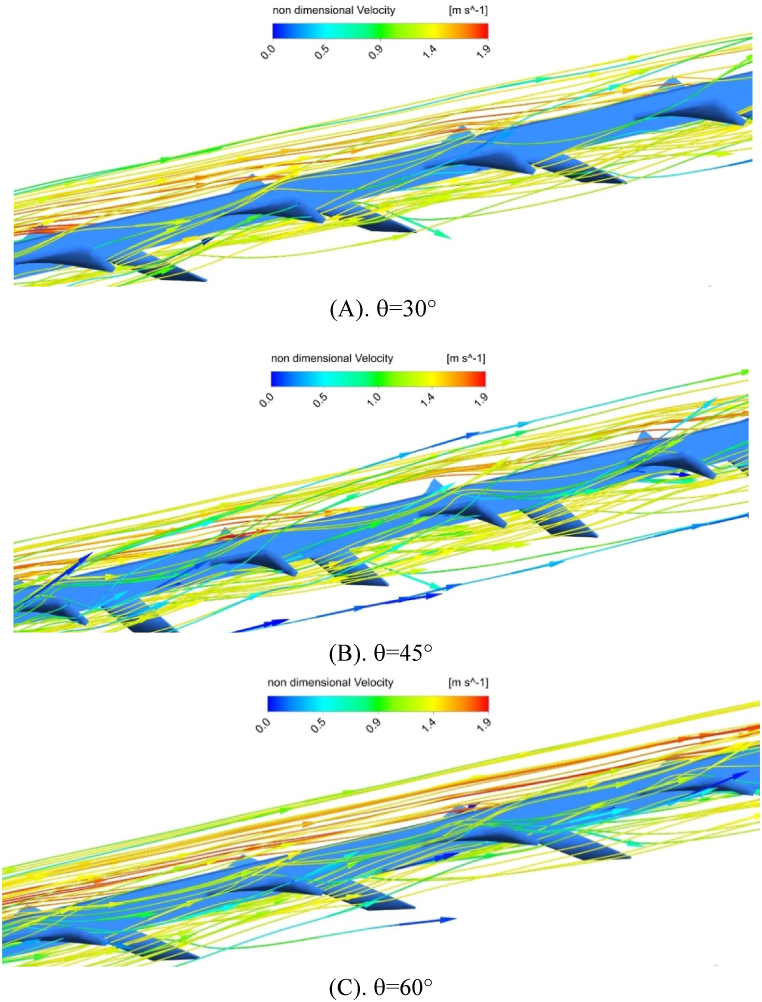
Flow velocity in twist angle of α = 45° and different angle of attack for pure water fluid in Reynolds number of 12000.

**Fig. 12:**
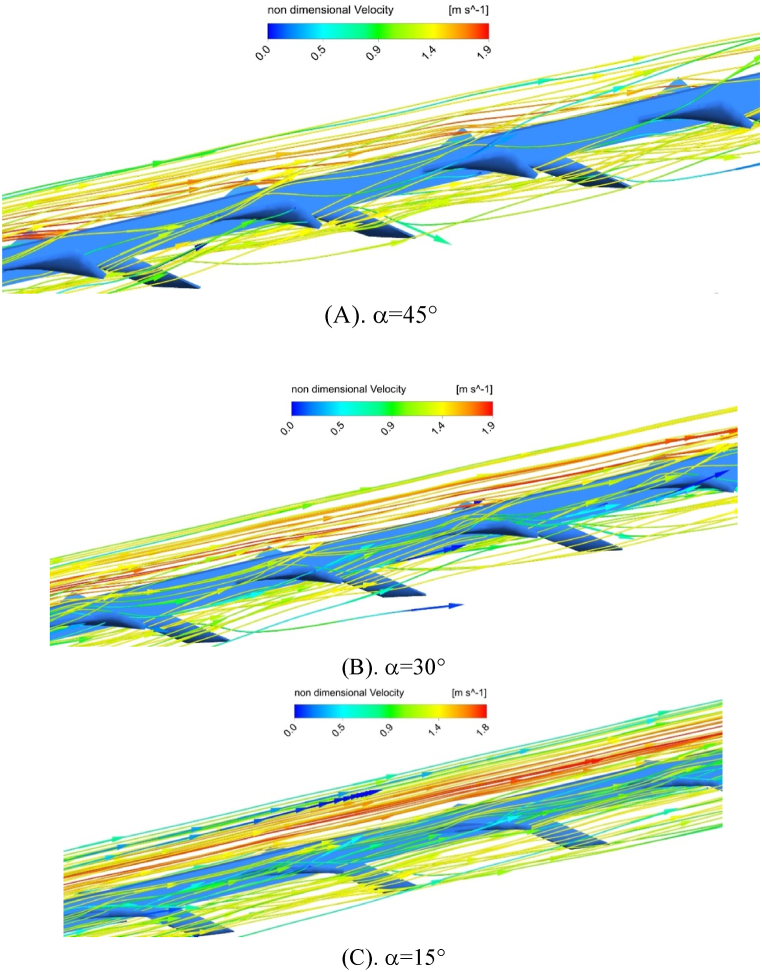
Flow velocity in the different twist angles and attack angle of θ = 30° for pure water fluid at Reynolds number of 12000.

Fig. 11 (A, B & C) represents flow velocity graphs for pure water fluid in Reynolds number of 12000, twist angle of 40° and angle of attack of θ = 30°(Fig. (11-A)), θ = 45° (Fig. (11-B)) and θ = 60° (Fig. (11-C)). The increase of winglet attack angle leads to the augmentation of flow deviation due to the location of winglets. According to this figure, the minimum deviation of flow is related to attack angle of 30° and the strongest velocity components are created in this attack angle due to the straight path of fluid. In attack angle of 60°, flow deviation and decrease of fluid momentum according to velocity level for each graph are obvious. Due to the existence of winglet with higher attack angle, the narrow cross section of flow causes fluid deviation and significantly affects the enhancement of fluid velocity. In Fig. 12 (A, B & C), flow velocity graphs are indicated in different twist angles (in α = 45° (Figure (12-A)), α = 30° (Figure (12-B)), and α = 15° (Figure (12-C))) and attack angle of θ = 30°. As in the previous figure, changes of twist angle of winglet will affect fluid flow domain and cause flow deviation from straight path and creation of secondary flows. Therefore, changes in flow velocity components in a twist angle of α = 45°, compared to α = 15°, are apparent.

## 6 Conclusion

In this study, twisted angles with different angle of attack are used for increasing flow circulation among winglets, flow mixture and fluid collision with hot surface of microchannel and heat transfer. This study uses water/Cu nanofluid with nanoparticle volume fractions of 0–4% in water as the working fluid due to the higher thermal conductivity. In this study, nanofluid's flow and heat transfer behavior are simulated by using 20 Aluminum winglets at the center of a rectangular microchannel with a length of 24 cm, which is affected by a constant heat flux of q" = 100 kw/m^2^. Results show that, by increasing twist angle of winglets, the amplitude of oscillations and created secondary vortexes are increased due to the sudden changes on fluid direction. Hence, the average Nusselt number and cooling performance of the hot surface are improved. By increasing the twist angle of the winglet, the flow mixture is reduced, which leads to the decrease in amplitude of created oscillations on cooling fluid moving on the hot surface. Therefore, the temperature of the hot surface increases with the enhancement of the twist angle of the winglet, which cannot improve the heat transfer process. Different twist angles of the winglet have similar quantitative values, indicating that the twist angle of the winglet has a significant effect on temperature distribution and preserves temperature gradients. Variations of winglet attack angle affect the intensity and power of created axial vortexes on flow direction and by decreasing the attack angle, created vortexes become stronger. Augmentation of the twist angle of winglet at higher Reynolds numbers and smaller angle of attack has the highest effect on heat transfer and Nusselt number enhancement. Increasing the angle of attack of the winglet can increase the friction coefficient up to 6 % for all the investigated situations. Changes in the twist angle on any fixed angle of attack also increase this parameter by 23 %. By decreasing the attack angle, the distribution of thermal boundary conditions causes better temperature distribution along the channel. Also, by increasing the twist angle of the winglet, heat transfer increases, which leads to the enhancement of fluid momentum depreciation and dominancy of the friction coefficient. According to the performance evaluation criterion (PEC), winglets lead to heat transfer enhancement. Figures of Nusselt number, friction coefficient, and performance evaluation criterion (PEC) reveal that adding solid nanoparticles to base fluid greatly affects thermal conductivity improvement; however, due to the increase of friction coefficient, performance evaluation criterion (PEC) reduces.

## Funding statement

This research did not receive any specific grant from funding agencies in the public, commercial, or not-for-profit sectors.

## Data availability statement

No data was used for the research described in the article.

## CRediT authorship contribution statement

**Mohammad Reza Tavakoli:** Writing – original draft, Resources, Project administration, Data curation, Conceptualization. **Omid Ali Akbari:** Writing – review & editing, Writing – original draft, Visualization, Validation, Supervision, Software, Resources, Project administration, Methodology, Investigation, Funding acquisition, Formal analysis, Data curation, Conceptualization. **Anoushiravan Mohammadian:** Visualization, Resources, Funding acquisition. **Farzad Pourfattah:** Writing – original draft, Validation, Project administration, Formal analysis.

## Declaration of competing interest

The authors declare that they have no known competing financial interests or personal relationships that could have appeared to influence the work reported in this paper.

**Table:** Nomenclature

C_p_	Heat capacity, J/kg K
D_h_	Hydrolic diameter, m
f	Friction factor
h	Convective heat transfer coefficient, W/m^2^.K
k	Thermal conductivity, W/m K
*Nu*	Nusselt number
q”	Thermal heat flux, W/m^2^
Re	Reynolds number
PEC	Performance Evaluation Criterion
s	Entropy, J/kg.K
T	Temperature, K
P	Pressure, pa
u	Velocity component a long the x, (m/s)
v	Velocity component a long the y, (m/s)
w	Velocity component along the z, (m/s)
Greek symbols	
ρ	Density, kg/m^3^
μ	Dynamic viscosity, Pa.s
φ	Nanoparticles volume fraction
**Super- and Sub-scripts**	
Ave	Average
b	Balk
f	Base fluid (Porewater)
nf	Nanofluid
p	Solid nanoparticles

## References

[1] Eiamsa-ard S., Wongcharee K., Eiamsa-ard P., Thianpong C. (2010). Heat transfer enhancement in a tube using delta-winglet twisted tape inserts. Appl. Therm. Eng..

[2] Wu J.M., Tao W.Q. (2007). Investigation on laminar convection heat transfer in fin-and-tube heat exchanger in aligned arrangement with longitudinal vortex generator from the viewpoint of field synergy principle. Appl. Therm. Eng..

[3] Colleoni A., Toutant A., Olalde G., Foucaunt J.M. (2013). Optimization of winglet vortex generators combined with riblets for wall/fluid heat exchanger enhancement. Appl. Therm. Eng..

[4] Kamboj R., Dhingra S., Singh G. (2014). CFD simulation of heat transfer enhancement by plain and curved winglet type vertex generators with punched holes. J Eng Res Gen Sci.

[5] Zhang Qiang, Wang Liang-Bi (2016). Numerical study of heat transfer enhancement by rectangular winglet vortex generator pair in a channel. Adv. Mech. Eng..

[6] Min C.H., Qi C.Y., Wang E.Y., Tian L.T., Qi Y.J. (2012). Numerical investigation of turbulent flow and heat transfer in a channel with novel longitudinal vortex generators. Int. J. Heat Mass Tran..

[7] Lu G., Zhou G. (2016). Numerical simulation on performances of plane and curved winglet type vortex generator pairs with punched holes. Int. J. Heat Mass Tran..

[8] Ghanbar Ali Sheikhzadeh, Faezeh Nejati Barzoki, Ali Akbar Abbasian Arani, Farzad Pourfattah, Wings shape effect on behavior of hybrid nanofluid inside a channel having vortex generator, Heat Mass Tran., 10.1007/s00231-018-2489-x.

[9] Caliskan S. (2014). Experimental investigation of heat transfer in a channel with new winglet-type vortex generators. Int. J. Heat Mass Tran..

[10] Lin Z.-M., Wang L.-B., Lin M., Dang W., Zhang Y.-H. (2017). Numerical study of the laminar flow and heat transfer characteristics in a tube inserting a twisted tape having parallelogram winglet vortex generators. Appl. Therm. Eng..

[11] Zhou G.B., Ye Q.L. (2012). Experimental investigations of thermal and flow characteristics of curved trapezoidal winglet type vortex generators. Appl. Therm. Eng..

[12] Lin Z.M., Liu C.P., Lin M., Wang L.B. (2015). Numerical study of flow and heat transfer enhancement of circular tube bank fin heat exchanger with curved delta-winglet vortex generators. Appl. Therm. Eng..

[13] Akbari O.A., Hassanzadeh Afrouzi H., Marzban A., Toghraie D., Malekzade H., Arabpour A. (2017). Investigation of volume fraction of nanoparticles effect and aspect ratio of the twisted tape in the tube. J. Therm. Anal. Calorim..

[14] Zaib A., Rashidi M.M., Chamkha A.J., Hattacharyya K.B. (2017). Numerical solution of second law analysis for MHD Casson nanofluid past a wedge with activation energy and binary chemical reaction. Int. J. Numer. Methods Heat Fluid Flow.

[15] Snoussi L., Ouerfelli N., Chesneau X., Chamkha A.J., Belgacem F.B.M., Guizani A. (2017). Natural convection heat transfer in a nanofluid filled U-shaped enclosures: numerical investigations. Heat Tran. Eng..

[16] Thumma T., Chamkha A., Sheri S.R. (2017). MHD natural convective flow of nanofluids past stationary and moving inclined porous plate considering temperature and concentration gradients with suction. Int. J. Numer. Methods Heat Fluid Flow.

[17] Chamkha A.J., Rashad M., Gorla R.S.R. (2014). Non-similar solutions for mixed convection along a wedge embedded in a porous medium saturated by a non-Newtonian nanofluid: natural convection dominated regime. Int. J. Numer. Methods Heat Fluid Flow.

[18] Chamkha A., Abbasbandy S., Rashad A.M. (2015). Non-Darcy natural convection flow for non-Newtonian nanofluid over cone saturated in porous medium with uniform heat and volume fraction fluxes. Int. J. Numer. Methods Heat Fluid Flow.

[19] Morshedzadeh E., Dunkenberger M.B., Nagle L., Ghasemi S., York L., Horn K. (2022). Tapping into community expertise: stakeholder engagement in the design process. Policy Design and Practice.

[20] Jones D., Ghasemi S., Gračanin D., Azab M. (2023).

[21] Hosseinnezhad R, Akbari OA, Hassanzadeh Afrouzi H, Biglarian M, Koveiti A, Toghraie D. The numerical study of heat transfer of turbulent nanofluid flow in a Tubular heat exchanger with twin twisted-Tapes inserts, J. Therm. Anal. Calorim., DOI 10.1007/s10973-017-6900-5.

[22] Andreozzi A., Manca O., Nardini S., Ricci D. (2016). Forced convection enhancement in channels with transversal ribs and nanofluids. Appl. Therm. Eng..

[23] Akbari O.A., Toghraie D., Karimipour A. (2016). Numerical simulation of heat transfer and turbulent flow of water nanofluids copper oxide in rectangular microchannel with semi attached rib. Adv. Mech. Eng..

[24] Alipour H., Karimipour A., Safaei M.R., Toghraie Semiromi D., Akbari O.A. (2017). Influence of T-semi attached rib on turbulent flow and heat transfer parameters of a silver-water nanofluid with different volume fractions in a three-dimensional trapezoidal microchannel. Physica E.

[25] Sheikholeslami M., Rashidi M.M., Ganji D.D. (2015). Numerical investigation of magnetic nanofluid forced convective heat transfer in existence of variable magnetic field using two phase model. J. Mol. Liq..

[26] Eiamsa-Ard S., Thianpong C., Eiamsa-Ard P. (2010). Turbulent heat transfer enhancement by counter/co-swirling flow in a tube fitted with twin twisted tapes. Exp. Therm. Fluid Sci..

[27] Promvonge P., Promthaisong P., Skullong S. (2020). Experimental and numerical heat transfer study of turbulent tube flow through discrete V-winglets. Int. J. Heat Mass Tran..

